# Measurement of Simple Reaction Time of the Cyclist in the Laboratory and Natural Environment Condition

**DOI:** 10.3390/s23083898

**Published:** 2023-04-11

**Authors:** Michał Żak, Grzegorz Mikrut, Grzegorz Sobota

**Affiliations:** 1 Institute of Sport Sciences, The Jerzy Kukuczka Academy of Physical Education in Katowice, 72A Mikołowska Street, 40-065 Katowice, Poland; michalzakwf@gmail.com; 2 Department of Management Theory, The Jerzy Kukuczka Academy of Physical Education in Katowice, 72A Mikołowska Street, 40-065 Katowice, Poland; g.mikrut@awf.katowice.pl

**Keywords:** simple reaction time, folic tactile sensor, measurement condition

## Abstract

The most commonly used reaction time tests within the athlete community require appropriate testing conditions and equipment, most frequently laboratory ones, which are not suitable for testing athletes in their natural environment and do not fully represent athletes’ natural capabilities and the influence of the surrounding environment. Therefore, this study’s goal is to compare the simple reaction times (SRTs) of cyclists during tests in laboratory conditions and in natural cycling surroundings. The young cyclists (55 participants) took part in the study. The SRT was measured in a quiet laboratory room with the use of the special device. During riding and standing with a bike outdoors, the necessary signal was captured and transmitted by a folic tactile sensor (FTS) and an extra intermediary circuit (both invented by our team member) connected to a muscle activity measurement system (Noraxon DTS Desktop, Scottsdale, AZ, USA). The results showed that external conditions significantly affect the SRT, with it being the longest when riding and the shortest if measured in an isolated laboratory room, but without an effect of gender. Typically, men have a shorter reaction time, but our result supports other observations, where people with an active lifestyle show no sex differentiation in SRT. The proposed FTS with an intermediary circuit allowed us to measure SRT with the use of non-dedicated equipment and avoid buying a new one for a single specific use.

## 1. Introduction

Cycling is an active form of traveling that can effectively increase the physical activity level of different aged people [1]. It helps to reduce the risk of overweight and obesity [2]. Cycling as a sport has been an Olympic discipline since 1896, and in the following years, more varieties were added to the Olympic program, such as track cycling, mountain biking and BMX [3]. Professional road cyclists cycle about 30,000 to 35,000 km during training and competition each year including up to 100 competition days. During cycling, many uncontrolled variables can affect performance such as weather conditions, altitude, wind direction and team strategy [4]. Moreover, terrain and competitive situations (individual riding or drafting at the back of a group of riders in a pack formation) can have an impact [5]. This requires the athletes to be very attentive and responsive to changing road conditions, where collisions with vehicles can be a significant safety issue for the cyclist. Moreover, collisions between vehicles and cyclists are not the only problem and falling off a bicycle for unexplained reasons, hitting an obstacle or going off the road can equally result in a serious risk of injury or death [6,7]. It seems, therefore, that reaction time and adequate response to a stimuli is one of the key elements in the safety of road traffic participants [2,8], and success in elite sport [9,10].

Reaction time (RT) is the elapsed time between the presentation of a sensory stimulus and the subsequent response [11]. We can distinguish a simple reaction and choice reaction time. The simple reaction time (SRT) is usually defined as the time it takes for an observer to detect one presented stimulus, e.g., a sound or a light signal. The choice reaction time (CRT) involves the decision process in which a subject needs to choose one correct response among several other stimulus. SRT is usually faster than CRT by about 100 ms [11], but there may be differences due to measurement and task conditions. Several studies show that men generally have shorter RTs than women [12,13,14]. Additionally, many studies agree that the average SRT decreases rapidly throughout childhood until adolescence and starts to increase progressively until old age but with lower intensity [13,15,16,17]. The human organism has many sensory inputs such as visual and auditory as well as tactile receptors. Depending on the environmental conditions and the task being performed, a specific stimulus may be dominant among others, for example, an auditory stimulus for an athlete at the start of a sprint run. In the literature, previous authors have reported that athletes have faster SRTs compared to non-athletes due to the fact that athletes often have to make the fastest possible decisions in repetitive sports situations [18]. RTs are evaluated by many devices and tests, starting with the simplest such as the ruler test [19], the finger tapping test [20], the use of computer software [21] or other special reaction time devices [22]. Surprisingly, the most commonly used RT tests within the athlete community [10,11], as well as those mentioned above, require appropriate testing conditions, most frequently laboratory ones, which are not suitable for testing athletes in their natural environment and do not fully represent athletes’ natural capabilities. Professional drivers, airplane pilots and maritime pilots can be tested in special traffic simulators on the road, air or sea because, unlike laboratory conditions, a number of other stimuli reach the body, most of them constituting information noise, from which the driver must perceive those relevant to driving and his safety and that of other road users [23]. To perform such observations, it is usually necessary to design and build or purchase dedicated measurement tools, which increases the cost of research. In many different areas, attempts are being made to keep conditions as close to natural as possible, reflecting the real environment and surroundings, when measuring or evaluating human capabilities [22,24,25].

Therefore, the primary study goal is to compare the reaction time of the cyclist during the test in laboratory conditions and in natural cycling surroundings. We hypothesize that the simple reaction time will depend on the cyclist’s environment during the measurement, and is shorter in laboratory conditions. The additional goal is to show how to use any other measuring devices for extra tasks with the help of a properly chosen sensor and, if necessary, an intermediary module.

## 2. Materials and Methods

Participants

Fifty-five cyclists represented by 21 girls and 34 boys (Table 1) from the Silesian cycling teams associated in Polish Cycling Federation participated in the study. The study was approved by the Institutional Ethics Committee and was conducted in accordance with the principles of the Declaration of Helsinki. Participants took part in the study only after their parents or legal guardians completed a written agreement to participate in the study.

Methods and Measurement protocol

**Figure 1:**
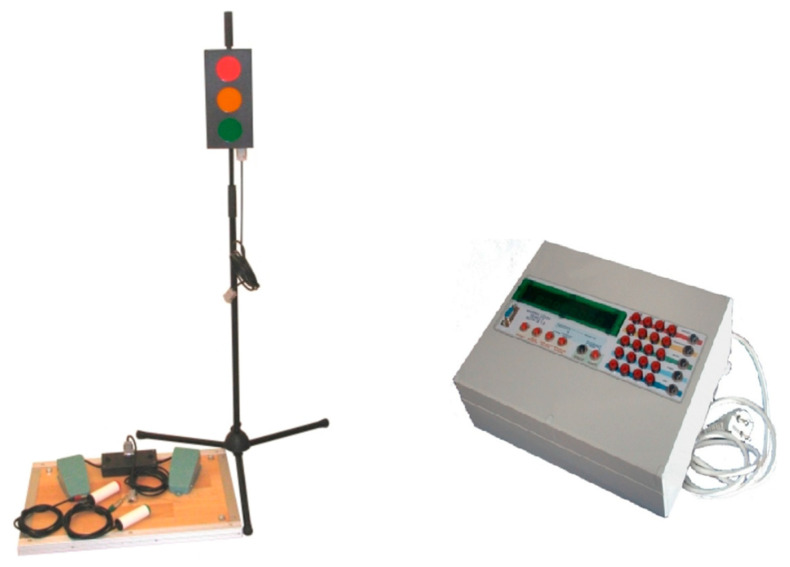
The MCZR/TB Response Time Meter 1.0 (ATB Info-Elektro, Ruda Śląska, Poland). Projector with the traffic lights and controls (push buttons and pedals) on the (**left**) and Central Unit on the (**right**).

Laboratory measurement:

The simple reaction time was measured with help the MCZR/TB Response Time Meter 1.0 (ATB Info-Elektro, Ruda Śląska, Poland). The measurement device (Figure 1) consisted of the Central Unit with control and programming panel, light stimulus signalling device and a set of sensory parts (buttons for hands and pedals for feet).

The Central Unit includes a set of buttons for operating the device as well as a display for reading the results and controlling the state of the unit. The stimulus signalling device and the stimulus receiving unit are connected to the Unit by cables plugged into the socket at the back of the device. Light stimulus device consists of a projector of three lights: red, orange and green imitating a traffic signal. The projector is placed on a metal tripod, which allows to set the height of the lights. During the evaluation, subject was seated in front of the lights (2 m apart), with arms placed freely along the trunk and the push button in the dominant hand. To determine the SRT, the subject’s task was to react immediately by pressing a button on the control in his hand as soon as the visual stimulus appeared. The reaction time was recorded and displayed on an electronic timer of the Central Unit. The procedure was repeated 5 times and mean values were taken for the statistical analysis.

Natural environment measurement:

The measurement of SRT was made while riding on the bicycle (dynamic conditions) and while standing with the bike (static conditions), during a sunny day with light breeze of wind (according to the Beaufort Scale) and a temperature of about 18 °C. During the tests, all subjects used one bicycle (mountain bike, aluminium frame, wheel size 26”), a frame size medium (M) suitable for youth. The height of the saddle was adjusted individually to the rider. There was no need to change gear, which was matched to easily reach the required speed.

**Figure 2:**
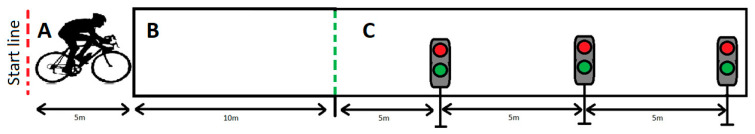
Spatial position of lights and areas during measurement in natural condition. A—speeding up area, B—correction velocity area, C—area with possible initiation of visual stimuli.

Participant cycled along a flat and straight path (with an asphalt surface) of 30 m length. First 5 m was for speeding up to 20 km/h, then 10 m to correct the velocity and keep it constant (verify with the speedometer on the handlebar), and next in every 5 m there were placed lights imitating traffic lights (three sets, Figure 2).

**Figure 3:**
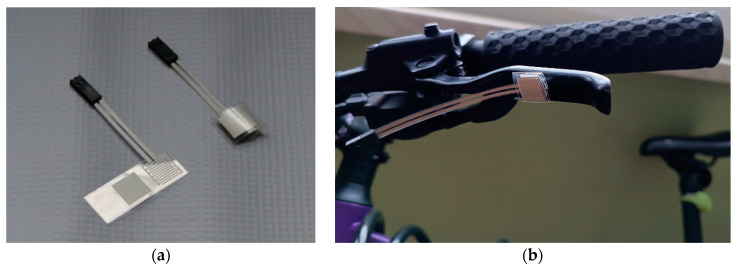
Folic tactile sensor (**a**) placed on the brake handle of the bike (**b**), patent PL 222119.B1 (author’s material).

**Figure 4:**
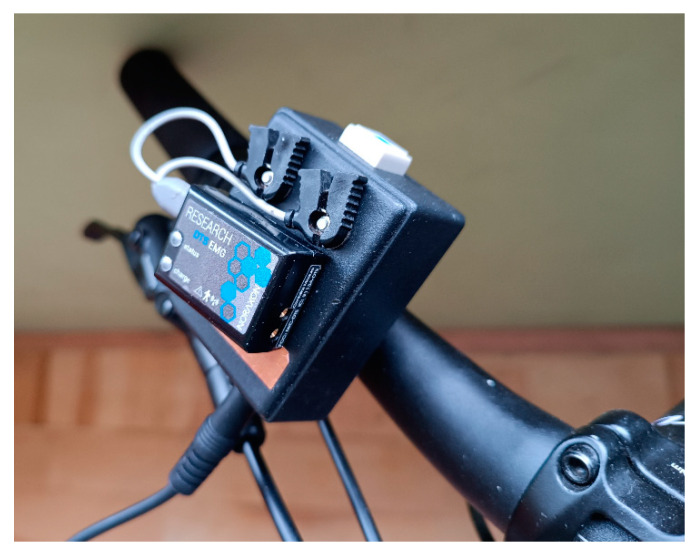
DTS EMG sensor with intermediary circuit–adopted Digital System for Determining Foot Contact with the Ground, Patent PL 222753.B1 (author’s material).

The traffic lights consisted of a tripod with adjustable height and two LED lights (white and red), positioned at the subject’s eye level. When the subject pressed the brake handle, a signal from the tactile sensor (folic tactile sensor, Patent PL 222119.B1 [26]) on the brake (Figure 3) was transmitted immediately to the data recording system using an appropriately adjusted intermediary circuit (it adopted Digital System for Determining Foot Contact with the Ground, Patent PL 222753.B1 [27]), coupled to the DTS EMG sensor (Noraxon, Scottsdale, AZ, USA) mounted on the bike (Figure 4) and next wirelessly with the DTS Desktop Receiver (Noraxon, USA) with a 1500 Hz sampling frequency.

The design assumption of the foil tactile sensor was to maximally simplify the construction and production process of the sensor, and thus reduce its price, thanks to which it will become possible to implement the concept of a disposable sensor. The disposable sensor eliminates the need for maintenance and disinfection after tests. The purchase prices of sensors available on the market are relatively high in relation to their durability. Production costs, in turn, are largely related to the degree of assembly of the structure. Therefore, a reduction in the price of the product can be obtained by minimizing the number of production stages while maintaining the precision of the sensor operation at the level offered by solutions available on the market.

**Figure 5:**
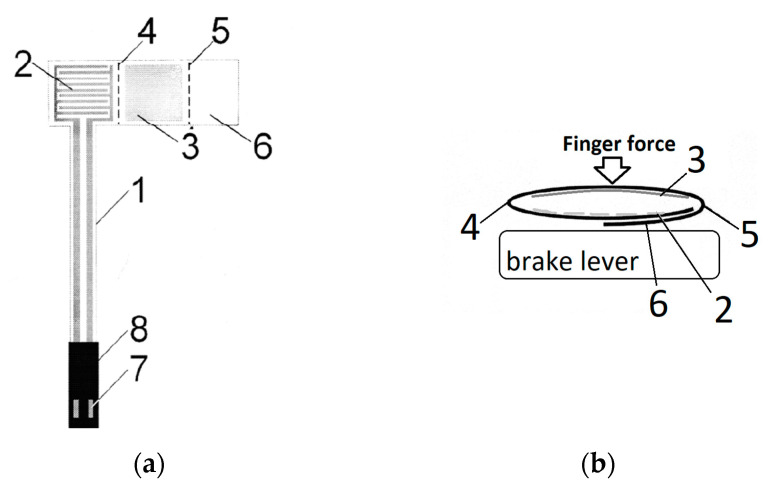
The schematic drawing of a folic tactile sensor (**a**) PL 222119.B1, where: 1—conductive tracks on the base layer, 2—electrodes, 3—jumper, 4—bending edge between electrodes and jumper, 5—bending edge between jumper and passive element, 6—passive element, 7—connector, 8—PCV connector housing, and ready to use sensor in current study setup (**b**) (author’s material).

The sensor intended for determining the contact of a limb or its part with an object or ground is characterized by the fact that it contains a single-element base layer made of non-conductive foil, divided by bending edges into three parts (Figure 5). The first one is a passive element ‘6’, the second one is a jumper ‘3’ placed on the middle one, and electrodes ‘2’ connected by conductive tracks ‘1’ with electrical terminals ‘7’ are placed on the last one. The base layer creates an elastic element on the bending edge located between the jumper and the passive element ‘5’ and on the bending edge located between the electrodes and the jumper ‘4’, enabling their electrical connection under pressure during contact limb/body part with the ground or other object. When the pressure on the sensor ends, it returns to its original shape, separating the jumper from the electrodes. The sensor uses the change in conductivity between the electrodes by shorting them with a jumper during contact of the limb with the ground or other objects. It can be powered by both DC and AC signals and used in systems where multi-layer contact force sensors (FSR—force sensing resistor) have been used as the standard, for example, to determine the contact of the limb with the ground during gait analysis.

The concept of a disposable sensor also takes into account its durability, which should ensure the stability of measurement parameters during the entire research cycle. The sensors were tested in terms of their use during gait tests, and high durability was obtained for the electrodes printed with silver paste, as they showed proper operation after a minimum of 10^3^ gait cycles at a cadence of 90 steps per minute. The standard protocol of electromyographic or optoelectronic gait tests typically require significantly fewer gait cycles.

The applied intermediary circuit in the form of digital circuit for determining the foot contact with the ground (PL 222753.B1, [27]), is characterized by it containing a sensor or a group of sensors connected, respectively, between the inputs of a single microcontroller or separate microcontrollers for each limb body point and a mass or a source of a high state corresponding to the microcontroller. This solution also includes electrical signal amplitude control circuits connected between the outputs of optional weight adders, and in the variant without weight adders, between the outputs of the appropriate microcontroller and differential inputs, for example, of a common electromyograph or analog-to-digital converter, which is integrated or connected, preferably wirelessly, to the recording system [27].

The entire process of recording and signal processing was managed by MyoResearch Clinical Applications software (Noraxon, Scottsdale, AZ, USA). The lights were connected with DTS Desktop Receiver with use of Analog Input DTS sensor (10 V range, Noraxon, Scottsdale, AZ, USA) to capture time of the light activation. The reaction time was determined from the time the stimulus signal (traffic light) appeared (the first sample of the rising edge for which the value was no less than 50% of the average value of the signal in the high state/level for minimum 10 ms) to the time when the high state of the brake signal appeared (the algorithm was the same as for the stimulus signal).

For the dynamic conditions (riding on the bike), the participant started on the command, achieved proper speed and when close to the lights (maximum 5 m before the first lights), at a random time, the light signal was activated by the researcher (the light signal appeared on each panel in the same time). The stimuli signal could be activated at any position of Area C (Figure 2) but not earlier than 5 m before the first lights and not later than 5 m before the last lights. The participant had to react as quickly as possible by pressing the brake handle when saw any light. The procedure was repeated 5 times and mean values were taken for the statistical analysis.

For the static conditions, the participant sat on the bicycle with one foot on the ground at a distance of 2 m from the middle panel with lights, and when any light stimulus appeared, the subject’s task was to react as quickly as possible by pressing the brake handle. The procedure was repeated 5 times and mean values were taken for the statistical analysis.

To avoid the influence of hand’s motion (from the handlebars to the brake lever) onto the SRT, the hand was already placed flat on the brake with the fingers extended so as not to press the lever in the direction of braking. To activate the brake, participants had only flex the fingers.

Statistical Analysis

The statistical analyses were conducted with the use of Statistica 9.0 software package (StatSoft, Inc., Tulsa, OK, USA). The data were examined for normality with Shapiro–Wilk test, for homogeneity of variances with Levene’s test. Assumption of homogeneity of variance was violated, hence the differences between conditions were confirmed by use of the non-parametric Anova Kruskal–Wallis test and to verify the differences between girls and boys an U Mann–Whitney test was adopted. Repeated measures tests could not be used because, for organizational reasons, not all subjects were tested under all conditions. Statistical significance level was set on *p* < 0.05.

Non-parametric R-Spearman’s correlation was made to analyse the correlation between age and SRT of the participants.

## 3. Results

The results showed no differences due to the sex of the subjects in each test reaction time condition. Only for the measurement in the natural environment during dynamic conditions was a trend towards statistical significance obtained (Z = 1.942, *p* = 0.0521, U Mann–Whitney test), indicating a faster reaction in boys. The global assessment of the differences in reaction time (without the sex effect) showed that all SRT measurement conditions were significantly different from each other (Kruskal–Wallis Test, H (2, 88) = 35.63, *p* < 0.0001). The shortest SRT was obtained by the subjects in the laboratory tests (median value 0.225 s) and the longest was during cycling (median value 0.348 s).

**Figure 6:**
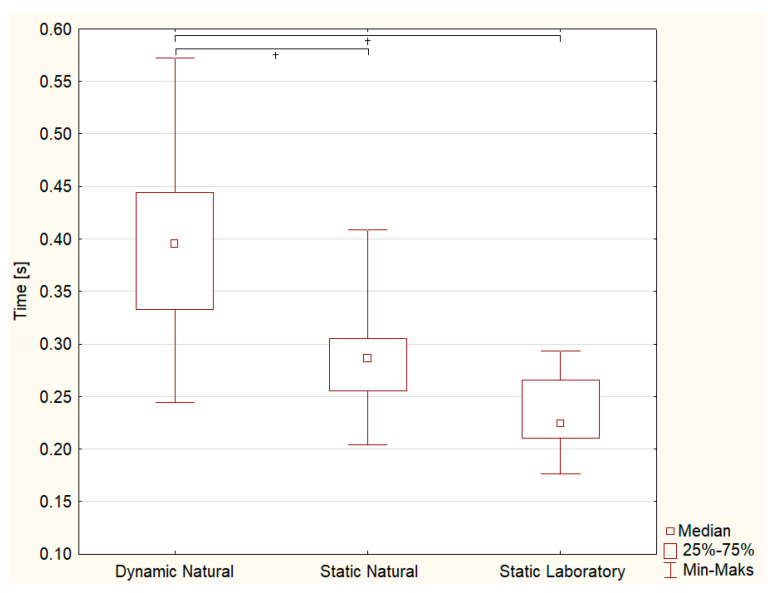
Box–whiskers plot of mean visual simple reaction time (s) under selected conditions in girls group (small box: median, box: first (25%) and third (75%) quartile, whiskers: minimum (Min) and maximum (Maks), cross (+): significant difference).

The analysis of the measurement conditions’ influence on SRT for girls showed that they had the longest reaction time in the natural environment while cycling (Figure 6, Dynamic Natural vs. Static Natural *p* = 0.036; Dynamic Natural vs. Static Laboratory *p* = 0.0007). The median values indicate a longer SRT when standing with the bike in relation to the laboratory conditions, but it was not a statistically significant difference (Figure 6, Static Natural vs. Static Laboratory *p* = 0.32).

**Figure 7:**
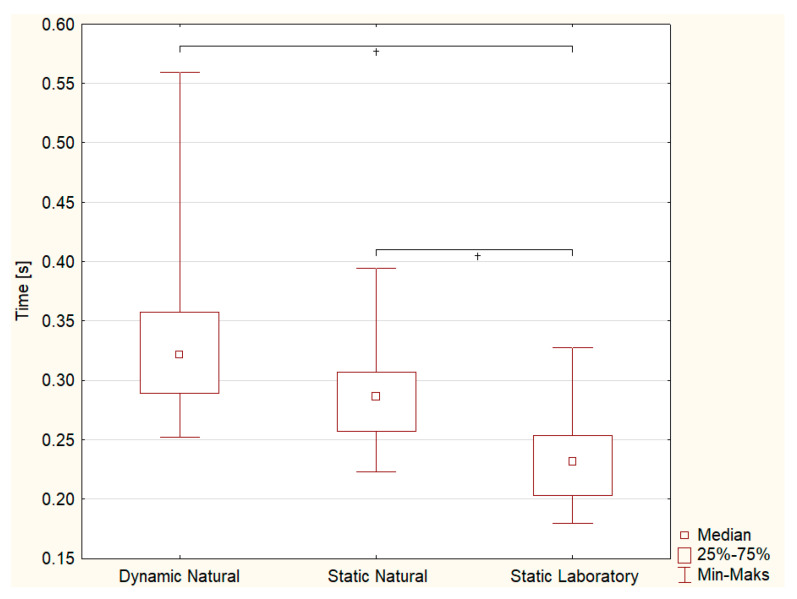
Box–whiskers plot of mean visual simple reaction time (s) under selected conditions in boys group (small box: median, box: first (25%) and third (75%) quartile, whiskers: minimum (Min) and maximum (Maks), cross (+): significant difference).

The analysis of the measurement conditions’ influence on SRT for boys showed that they had the significantly shortest reaction time in the laboratory condition (Figure 7, Dynamic Natural vs. Static Laboratory *p* < 0.0001; Static Natural vs. Static Laboratory *p* = 0.039). The median values indicate a longer SRT when riding in relation to standing with the bike, but it did not differ significantly (Figure 7, Dynamic Natural vs. Static Natural *p* = 0.071).

Pearson correlation results (Table 2) showed a significant relationship between reaction time and age (*p* < 0.05) under static laboratory conditions. It was shown that the reaction time decreases with age, which illustrates a negative correlation between variables (r = −0.725). In addition, a weak significant relationship (*p* < 0.05) was shown under natural dynamic conditions (r = −0.363). In contrast, statistically significant correlations between age and reaction time were not found in static natural conditions (Table 2).

## 4. Discussion

In a constantly changing environment, the ability to react quickly is essential to virtually every human. However, within the athlete community, it seems to be even more pronounced. It is not only related to achieving good sports results [9,10,28,29], but also affects the safety of the athlete, especially those who use public space in their training, including public roads [2,8]. Gender is a factor differentiating SRT in favour of men [12,13,14], but the results of this study indicate no significant differences in SRT for all observed conditions, apart from a trend towards significance during cycling (boys revealed shorter SRTs than girls, *p* = 0.054). Such characteristics are consistent with the observations of Jain et al. [30], who did not observe gender differences in regularly exercising medical students, while reaction time was significantly different according to sex in the sedentary lifestyle group. It seems, therefore, that a high level of physical activity may blur gender differences in a simple reaction to a visual stimulus [30]. Various external stimuli, e.g., heat, light intensity [31], noise [32] and other external and internal factors (e.g., mental fatigue [33]) can significantly affect SRT. In laboratory conditions, the boys obtained the shortest simple reaction times, and the differences between the dynamic and static conditions during the study in the natural external environment were not statistically confirmed (Figure 7). It seems, therefore, that the influence of external conditions affects the behaviour of boys more than girls, who had a significantly longer SRT during cycling when compared to static conditions, regardless of the type of surrounding environment (Figure 6). Perhaps girls need more attention when cycling, as previous authors reported that attention level strongly influences SRT [34,35], and it is also influenced by tasks that require higher body stability [36]. Riding on a bike is not just about maintaining balance but also controlling the direction of motion, speed and eyes–hands coordination. Each of these elements demands some time for information processing, and there is not enough left for time reaction, so the results are worse [32,34].

The negative effect of age on the SRT value, when measured in laboratory conditions (Table 2, r = −0.725), was consistent with other studies [13,15,16]. However, in the natural environment, only a weak trend was obtained for static conditions (standing with a bike) and there was no correlation for dynamic conditions (during riding). This may indicate an additional influence of athlete’s experience, which dominates over the influence of a participant’s age.

A technical solution using a proprietary tactile sensor (Patent PL 222119.B1 [26]) was presented, which can be used in various applications. The possibility of its use was determined by the size and lack of interference from the external environment for correct operation and sufficient functionality in this task. The key requirement for the measurement setup was wireless data transmission between the moving cyclist and the stationary measurement system, synchronously recording the signals of visual stimulation and the response of the subject. For this purpose, the wireless muscle activity analysis system DTS Desktop Noraxon (Noraxon USA) was used, which is usually used to assess the work of the muscular system [37,38]. Using the information on the characteristics of the measurement signals that can be recorded on our actual equipment, it is possible to prepare a module that adjusts (Patent PL 222753.B1 [27]) the signal from any sensor to the inputs of the measurement system [39]. Therefore, the module (Figure 4) generates an artificial bioelectric signal of the muscle when the tactile sensor is active (in this paper—the brake lever is pressed). Thanks to this, there is no need for a new measurement set dedicated to only one specific application, which often happens in scientific research. It is a very common phenomenon that after the completion of a specific research project, the new apparatus or measurement system produced for its needs is moved to the warehouse and is no longer used. After 2–3 years it becomes a waste because new technologies dominate. We propose the solution, which reduces research costs and is in line with the policy of environmental protection and saving the Earth’s material resources [40,41].

There are a few limitations of this study according to the simple reaction time results. The first limitation was the age of the subjects—the youngest of them was 11 years old. It is possible that their nervous system is not yet mature enough, which may result in a longer reaction time than we expected based on the literature review [15,16]. The results of the correlation analysis between age and SRT indicate just such an effect; however, it was more important for us to show the changes in SRT due to the conditions in which the assessment was made. The second limitation was carrying out the measurement in conditions very close to the ‘cyclists environment’ but not during normal road traffic. The external conditions were the same as when driving on a public road: a similar type and intensity of various noises, wind, lighting and temperature (the measuring track was located in the vicinity of the main road). The only difference was the lack of cars on the measurement section, which was necessary to maintain the safety of the surveyed athletes. The third limitation was to use only one specific speed, and our future step will be to determine how the speed level influences the reaction time on visual stimuli.

## 5. Conclusions

The results of this study indicate no significant differences in SRT for all test conditions due to gender. External conditions significantly affect the SRT, with the longest when riding and the shortest if measured in an isolated laboratory room. The negative correlation of age on the SRT value was achieved for laboratory conditions and only a weak trend was obtained for static conditions (standing with the bike) and no correlation for dynamic conditions (during riding) in the natural environment. The proposed folic tactile sensor with the additional intermediary circuit allowed us to measure SRT with the use of non-dedicated equipment and lower the cost of the study.

## Figures and Tables

**Table 1:** Characteristic of study group (values are mean +/− STD).

	Age (Years)	Body Height (cm)	Body Mass (kg)
Boys	13.4 ± 1.53	165.4 ± 10.77	48.7 ± 12.63
Girls	12.6 ± 1.58	165.2 ± 5.35	47.8 ± 11.13

**Table 2:** The correlation coefficient of age and simple reaction time under selected measurement conditions.

Measurement Conditions	Age—SRT Correlation	
	r	*p*
Static Laboratory	−0.725	0.0002 *
Dynamic Natural	−0.363	0.034 *
Static Natural	−0.114	0.52

* significant correlation at *p* < 0.05, r—correlation coefficient.

## Data Availability

The data presented in this study are available on request from the corresponding author. The data are not publicly available due to privacy.
